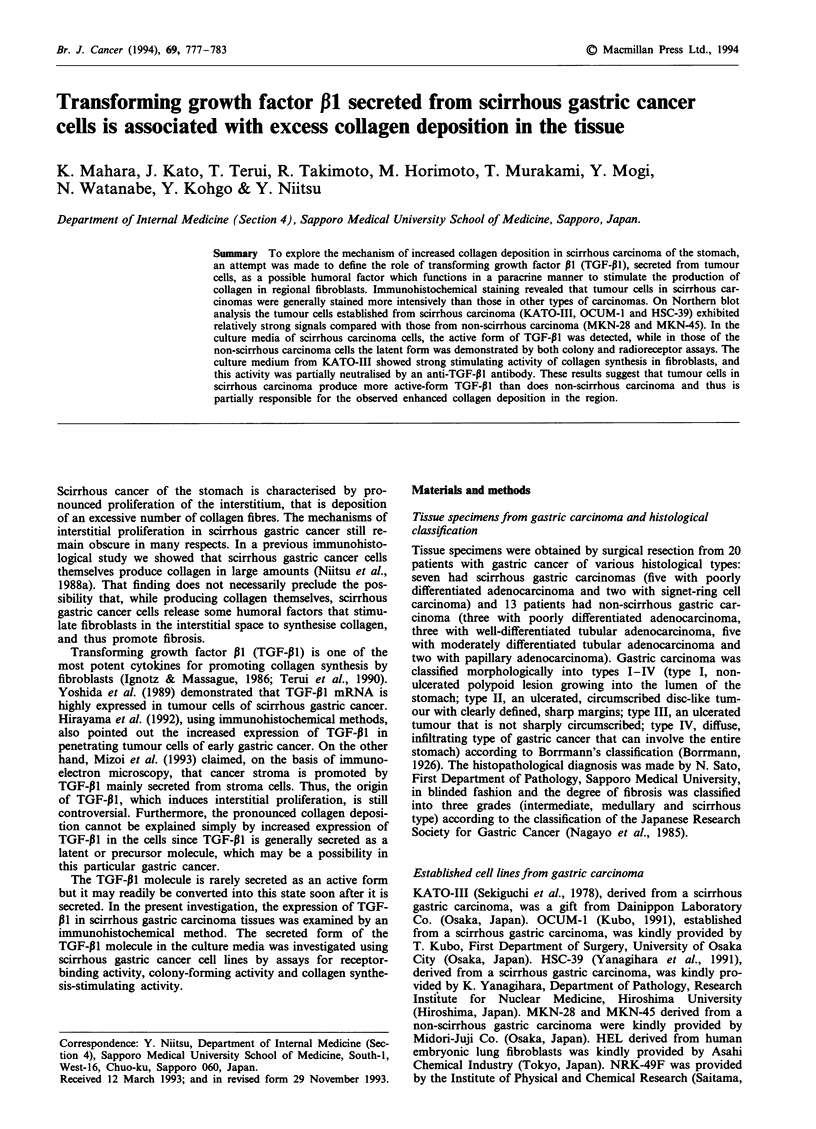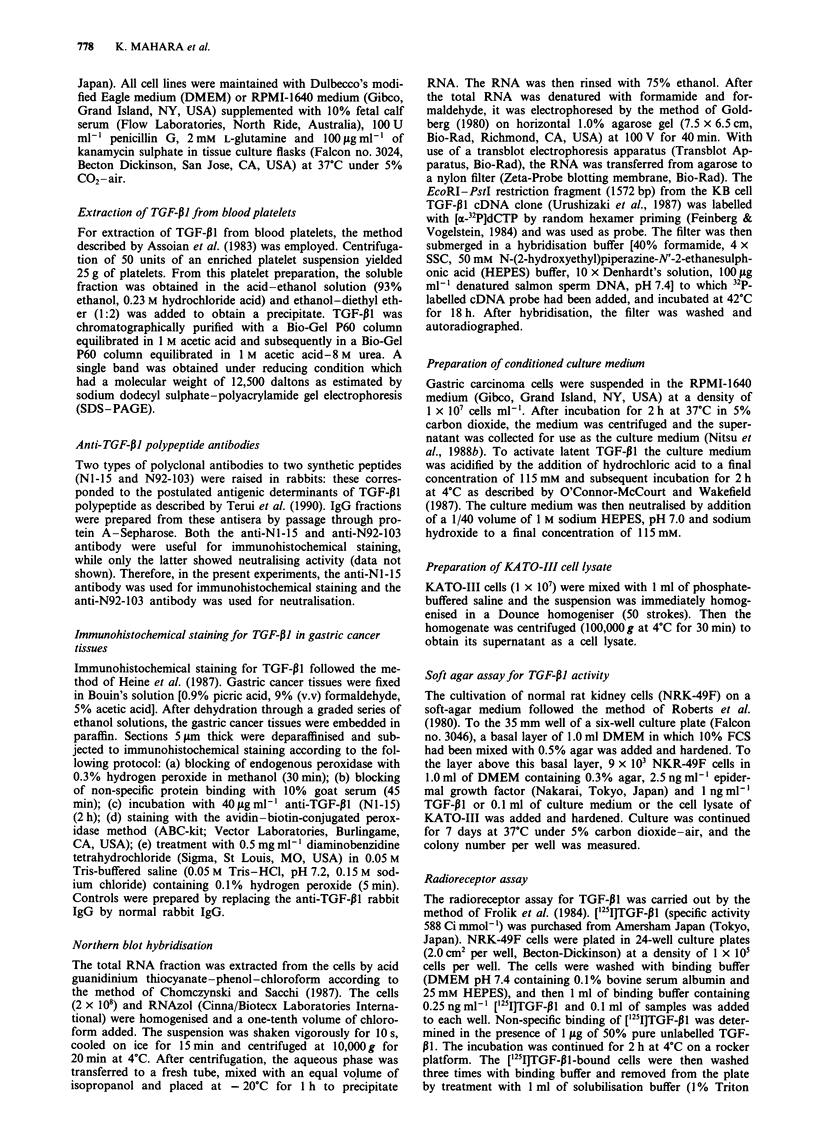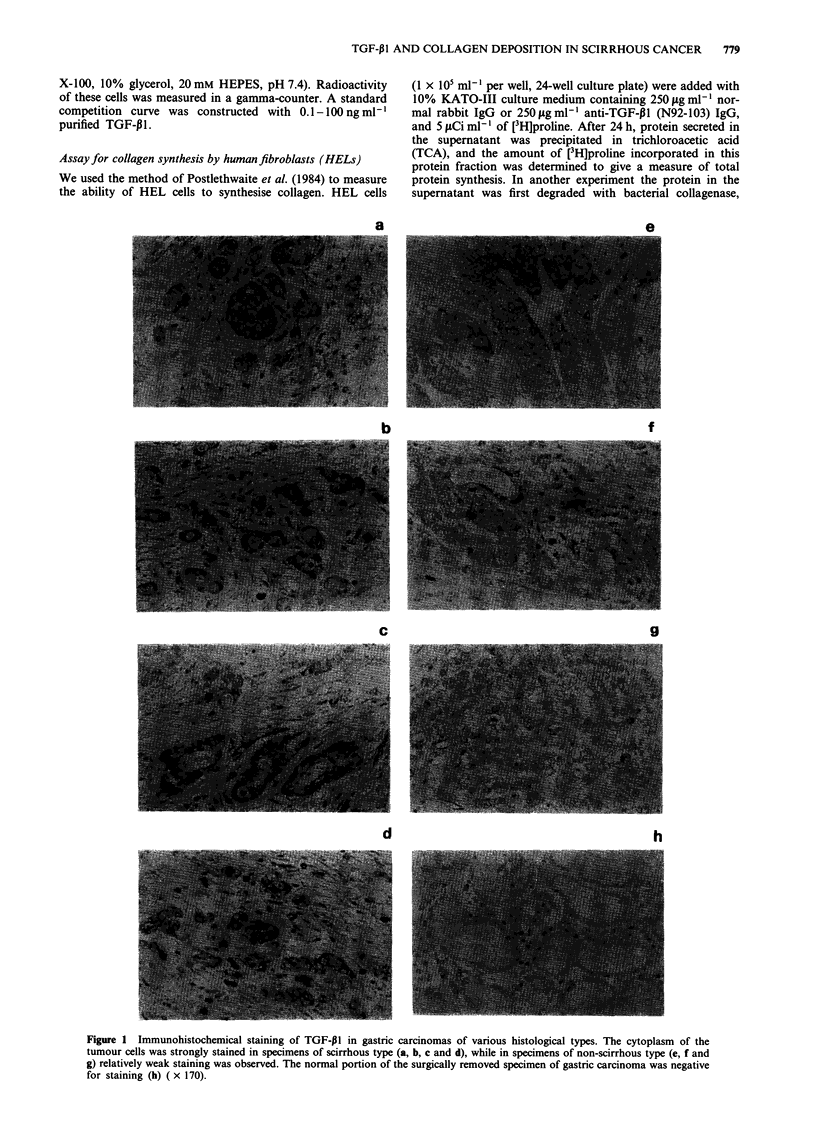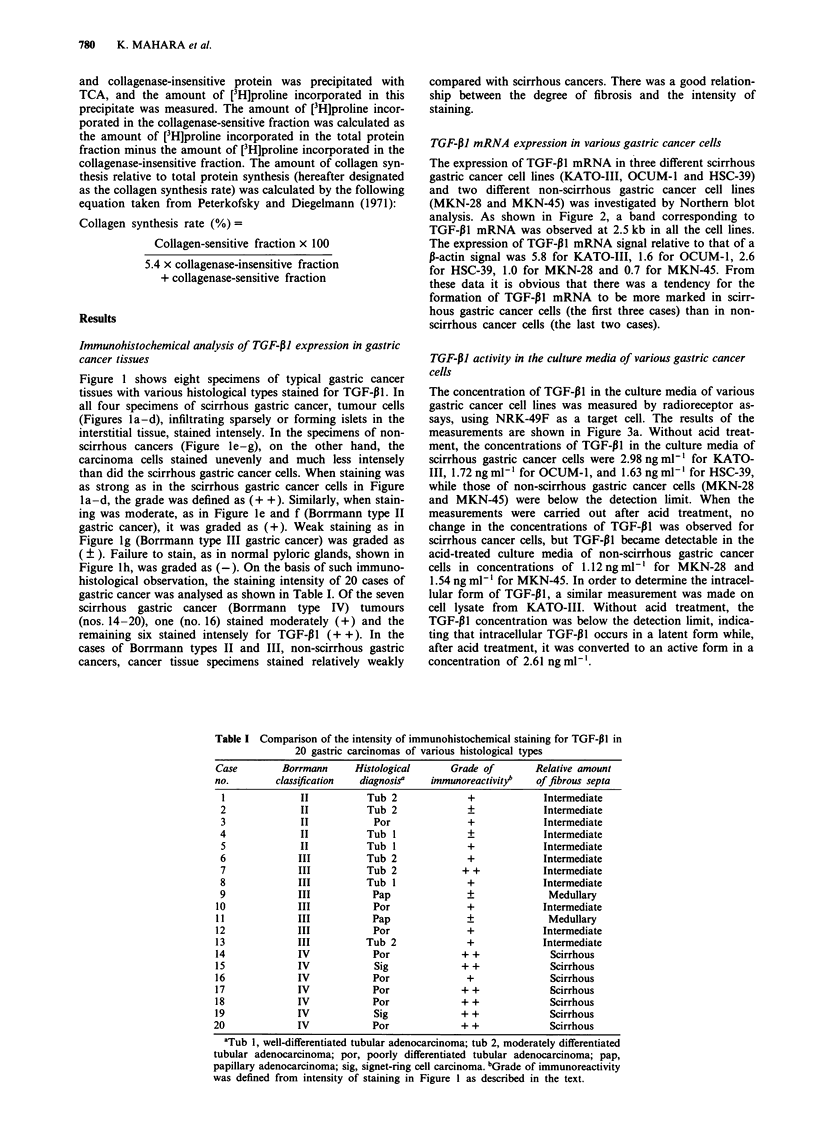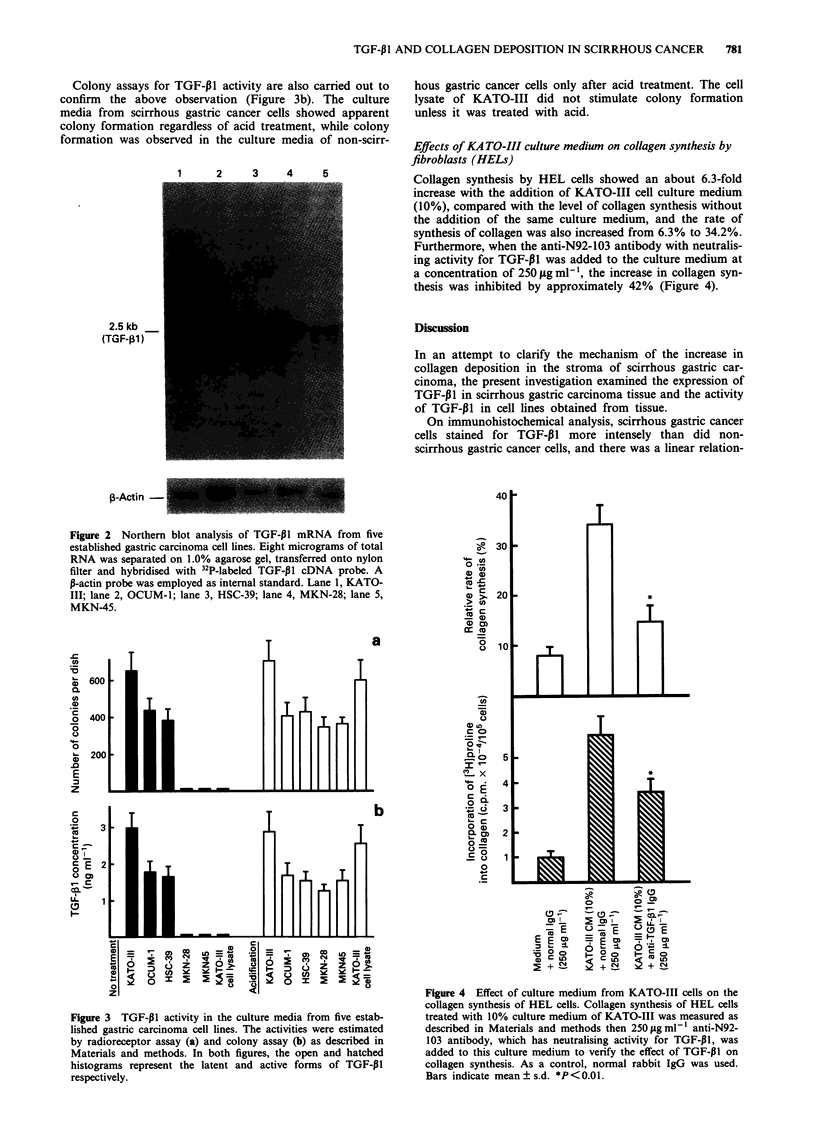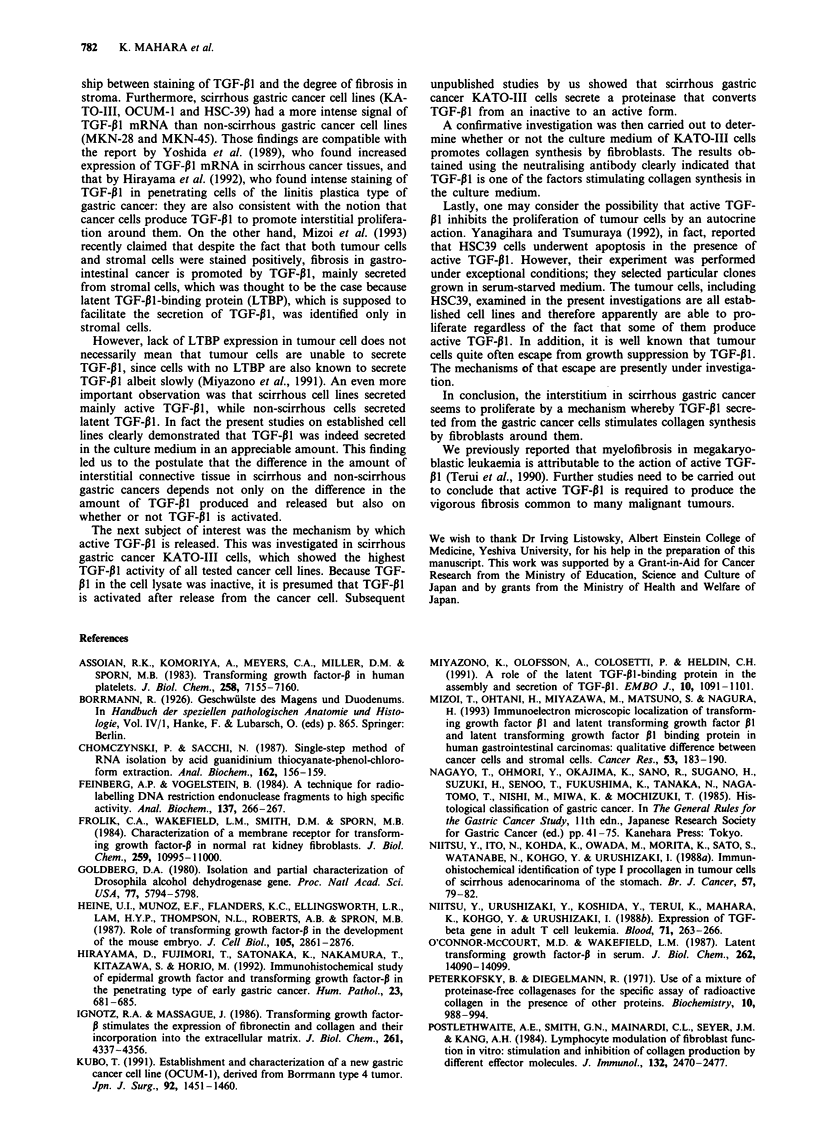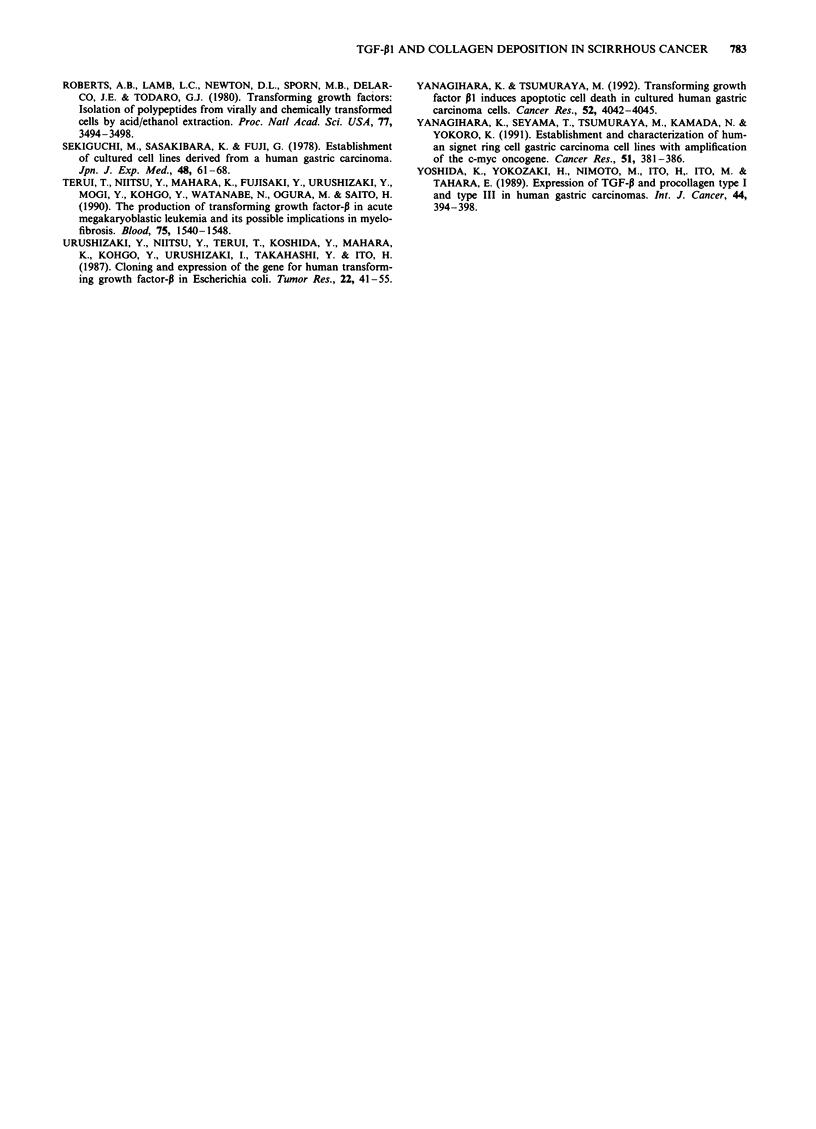# Transforming growth factor beta 1 secreted from scirrhous gastric cancer cells is associated with excess collagen deposition in the tissue.

**DOI:** 10.1038/bjc.1994.147

**Published:** 1994-04

**Authors:** K. Mahara, J. Kato, T. Terui, R. Takimoto, M. Horimoto, T. Murakami, Y. Mogi, N. Watanabe, Y. Kohgo, Y. Niitsu

**Affiliations:** Department of Internal Medicine (Section 4), Sapporo Medical University School of Medicine, Japan.

## Abstract

**Images:**


*Br. J. Cancer* (1994), **69**, 777–783 © Macmillan Press Ltd., 1994

### Transforming growth factor β1 secreted from scirrhous gastric cancer cells is associated with excess collagen deposition in the tissue

K. Mahara, J. Kato, T. Terui, R. Takimoto, M. Horimoto, T. Murakami, Y. Mogi, N. Watanabe, Y. Kohgo & Y. Niitsu

*Department of Internal Medicine (Section 4), Sapporo Medical University School of Medicine, Sapporo, Japan.*

**Summary** To explore the mechanism of increased collagen deposition in scirrhous carcinoma of the stomach, an attempt was made to define the role of transforming growth factor β1 (TGF-β1), secreted from tumour cells, as a possible humoral factor which functions in a paracrine manner to stimulate the production of collagen in regional fibroblasts. Immunohistochemical staining revealed that tumour cells in scirrhous carcinomas were generally stained more intensively than those in other types of carcinomas. On Northern blot analysis the tumour cells established from scirrhous carcinoma (KATO-III, OCUM-1 and HSC-39) exhibited relatively strong signals compared with those from non-scirrhous carcinoma (MKN-28 and MKN-45). In the culture media of scirrhous carcinoma cells, the active form of TGF-β1 was detected, while in those of the non-scirrhous carcinoma cells the latent form was demonstrated by both colony and radioreceptor assays. The culture medium from KATO-III showed strong stimulating activity of collagen synthesis in fibroblasts, and this activity was partially neutralised by an anti-TGF-β1 antibody. These results suggest that tumour cells in scirrhous carcinoma produce more active-form TGF-β1 than does non-scirrhous carcinoma and thus is partially responsible for the observed enhanced collagen deposition in the region.

Scirrhous cancer of the stomach is characterised by pronounced proliferation of the interstitium, that is deposition of an excessive number of collagen fibres. The mechanisms of interstitial proliferation in scirrhous gastric cancer still remain obscure in many respects. In a previous immunohistological study we showed that scirrhous gastric cancer cells themselves produce collagen in large amounts (Niitsu *et al.*, 1988a). That finding does not necessarily preclude the possibility that, while producing collagen themselves, scirrhous gastric cancer cells release some humoral factors that stimulate fibroblasts in the interstitial space to synthesise collagen, and thus promote fibrosis.

Transforming growth factor β1 (TGF-β1) is one of the most potent cytokines for promoting collagen synthesis by fibroblasts (Ignotz & Massague, 1986; Terui *et al.*, 1990). Yoshida *et al.* (1989) demonstrated that TGF-β1 mRNA is highly expressed in tumour cells of scirrhous gastric cancer. Hirayama *et al.* (1992), using immunohistochemical methods, also pointed out the increased expression of TGF-β1 in penetrating tumour cells of early gastric cancer. On the other hand, Mizoi *et al.* (1993) claimed, on the basis of immunoelectron microscopy, that cancer stroma is promoted by TGF-β1 mainly secreted from stroma cells. Thus, the origin of TGF-β1, which induces interstitial proliferation, is still controversial. Furthermore, the pronounced collagen deposition cannot be explained simply by increased expression of TGF-β1 in the cells since TGF-β1 is generally secreted as a latent or precursor molecule, which may be a possibility in this particular gastric cancer.

The TGF-β1 molecule is rarely secreted as an active form but it may readily be converted into this state soon after it is secreted. In the present investigation, the expression of TGF-β1 in scirrhous gastric carcinoma tissues was examined by an immunohistochemical method. The secreted form of the TGF-β1 molecule in the culture media was investigated using scirrhous gastric cancer cell lines by assays for receptor-binding activity, colony-forming activity and collagen synthesis-stimulating activity.

Correspondence: Y. Niitsu, Department of Internal Medicine (Section 4), Sapporo Medical University School of Medicine, South-1, West-16, Chuo-ku, Sapporo 060, Japan.
Received 12 March 1993; and in revised form 29 November 1993.

### Materials and methods

### *Tissue specimens from gastric carcinoma and histological classification*

Tissue specimens were obtained by surgical resection from 20 patients with gastric cancer of various histological types: seven had scirrhous gastric carcinomas (five with poorly differentiated adenocarcinoma and two with signet-ring cell carcinoma) and 13 patients had non-scirrhous gastric carcinoma (three with poorly differentiated adenocarcinoma, three with well-differentiated tubular adenocarcinoma, five with moderately differentiated tubular adenocarcinoma and two with papillary adenocarcinoma). Gastric carcinoma was classified morphologically into types I–IV (type I, non-ulcerated polypoid lesion growing into the lumen of the stomach; type II, an ulcerated, circumscribed disc-like tumour with clearly defined, sharp margins; type III, an ulcerated tumour that is not sharply circumscribed; type IV, diffuse, infiltrating type of gastric cancer that can involve the entire stomach) according to Borrmann's classification (Borrmann, 1926). The histopathological diagnosis was made by N. Sato, First Department of Pathology, Sapporo Medical University, in blinded fashion and the degree of fibrosis was classified into three grades (intermediate, medullary and scirrhous type) according to the classification of the Japanese Research Society for Gastric Cancer (Nagayo *et al.*, 1985).

### *Established cell lines from gastric carcinoma*

KATO-III (Sekiguchi *et al.*, 1978), derived from a scirrhous gastric carcinoma, was a gift from Dainippon Laboratory Co. (Osaka, Japan). OCUM-1 (Kubo, 1991), established from a scirrhous gastric carcinoma, was kindly provided by T. Kubo, First Department of Surgery, University of Osaka City (Osaka, Japan). HSC-39 (Yanagihara *et al.*, 1991), derived from a scirrhous gastric carcinoma, was kindly provided by K. Yanagihara, Department of Pathology, Research Institute for Nuclear Medicine, Hiroshima University (Hiroshima, Japan). MKN-28 and MKN-45 derived from a non-scirrhous gastric carcinoma were kindly provided by Midori-Juji Co. (Osaka, Japan). HEL derived from human embryonic lung fibroblasts was kindly provided by Asahi Chemical Industry (Tokyo, Japan). NRK-49F was provided by the Institute of Physical and Chemical Research (Saitama,

Japan). All cell lines were maintained with Dulbecco's modified Eagle medium (DMEM) or RPMI-1640 medium (Gibco, Grand Island, NY, USA) supplemented with 10% fetal calf serum (Flow Laboratories, North Ride, Australia), 100 U $ml^{-1}$ penicillin G, 2 mM L-glutamine and 100 $\mu g\ ml^{-1}$ of kanamycin sulphate in tissue culture flasks (Falcon no. 3024, Becton Dickinson, San Jose, CA, USA) at 37°C under 5% $CO_2$–air.

*Extraction of TGF-β1 from blood platelets*

For extraction of TGF-β1 from blood platelets, the method described by Assoian *et al.* (1983) was employed. Centrifugation of 50 units of an enriched platelet suspension yielded 25 g of platelets. From this platelet preparation, the soluble fraction was obtained in the acid–ethanol solution (93% ethanol, 0.23 M hydrochloride acid) and ethanol–diethyl ether (1:2) was added to obtain a precipitate. TGF-β1 was chromatographically purified with a Bio-Gel P60 column equilibrated in 1 M acetic acid and subsequently in a Bio-Gel P60 column equilibrated in 1 M acetic acid–8 M urea. A single band was obtained under reducing condition which had a molecular weight of 12,500 daltons as estimated by sodium dodecyl sulphate–polyacrylamide gel electrophoresis (SDS–PAGE).

*Anti-TGF-β1 polypeptide antibodies*

Two types of polyclonal antibodies to two synthetic peptides (N1-15 and N92-103) were raised in rabbits: these corresponded to the postulated antigenic determinants of TGF-β1 polypeptide as described by Terui *et al.* (1990). IgG fractions were prepared from these antisera by passage through protein A–Sepharose. Both the anti-N1-15 and anti-N92-103 antibody were useful for immunohistochemical staining, while only the latter showed neutralising activity (data not shown). Therefore, in the present experiments, the anti-N1-15 antibody was used for immunohistochemical staining and the anti-N92-103 antibody was used for neutralisation.

*Immunohistochemical staining for TGF-β1 in gastric cancer tissues*

Immunohistochemical staining for TGF-β1 followed the method of Heine *et al.* (1987). Gastric cancer tissues were fixed in Bouin's solution [0.9% picric acid, 9% (v.v) formaldehyde, 5% acetic acid]. After dehydration through a graded series of ethanol solutions, the gastric cancer tissues were embedded in paraffin. Sections 5 μm thick were deparaffinised and subjected to immunohistochemical staining according to the following protocol: (a) blocking of endogenous peroxidase with 0.3% hydrogen peroxide in methanol (30 min); (b) blocking of non-specific protein binding with 10% goat serum (45 min); (c) incubation with 40 $\mu g\ ml^{-1}$ anti-TGF-β1 (N1-15) (2 h); (d) staining with the avidin–biotin-conjugated peroxidase method (ABC-kit; Vector Laboratories, Burlingame, CA, USA); (e) treatment with 0.5 mg $ml^{-1}$ diaminobenzidine tetrahydrochloride (Sigma, St Louis, MO, USA) in 0.05 M Tris-buffered saline (0.05 M Tris–HCl, pH 7.2, 0.15 M sodium chloride) containing 0.1% hydrogen peroxide (5 min). Controls were prepared by replacing the anti-TGF-β1 rabbit IgG by normal rabbit IgG.

*Northern blot hybridisation*

The total RNA fraction was extracted from the cells by acid guanidinium thiocyanate–phenol–chloroform according to the method of Chomczynski and Sacchi (1987). The cells ($2 \times 10^8$) and RNAzol (Cinna/Biotecx Laboratories International) were homogenised and a one-tenth volume of chloroform added. The suspension was shaken vigorously for 10 s, cooled on ice for 15 min and centrifuged at 10,000 *g* for 20 min at 4°C. After centrifugation, the aqueous phase was transferred to a fresh tube, mixed with an equal volume of isopropanol and placed at −20°C for 1 h to precipitate RNA. The RNA was then rinsed with 75% ethanol. After the total RNA was denatured with formamide and formaldehyde, it was electrophoresed by the method of Goldberg (1980) on horizontal 1.0% agarose gel (7.5 × 6.5 cm, Bio-Rad, Richmond, CA, USA) at 100 V for 40 min. With use of a transblot electrophoresis apparatus (Transblot Apparatus, Bio-Rad), the RNA was transferred from agarose to a nylon filter (Zeta-Probe blotting membrane, Bio-Rad). The *Eco*RI–*Pst*I restriction fragment (1572 bp) from the KB cell TGF-β1 cDNA clone (Urushizaki *et al.*, 1987) was labelled with [α-$^{32}$P]dCTP by random hexamer priming (Feinberg & Vogelstein, 1984) and was used as probe. The filter was then submerged in a hybridisation buffer [40% formamide, 4 × SSC, 50 mM N-(2-hydroxyethyl)piperazine-*N*′-2-ethanesulphonic acid (HEPES) buffer, 10 × Denhardt's solution, 100 μg $ml^{-1}$ denatured salmon sperm DNA, pH 7.4] to which $^{32}$P-labelled cDNA probe had been added, and incubated at 42°C for 18 h. After hybridisation, the filter was washed and autoradiographed.

*Preparation of conditioned culture medium*

Gastric carcinoma cells were suspended in the RPMI-1640 medium (Gibco, Grand Island, NY, USA) at a density of $1 \times 10^7$ cells $ml^{-1}$. After incubation for 2 h at 37°C in 5% carbon dioxide, the medium was centrifuged and the supernatant was collected for use as the culture medium (Nitsu *et al.*, 1988*b*). To activate latent TGF-β1 the culture medium was acidified by the addition of hydrochloric acid to a final concentration of 115 mM and subsequent incubation for 2 h at 4°C as described by O'Connor-McCourt and Wakefield (1987). The culture medium was then neutralised by addition of a 1/40 volume of 1 M sodium HEPES, pH 7.0 and sodium hydroxide to a final concentration of 115 mM.

*Preparation of KATO-III cell lysate*

KATO-III cells ($1 \times 10^7$) were mixed with 1 ml of phosphate-buffered saline and the suspension was immediately homogenised in a Dounce homogeniser (50 strokes). Then the homogenate was centrifuged (100,000 *g* at 4°C for 30 min) to obtain its supernatant as a cell lysate.

*Soft agar assay for TGF-β1 activity*

The cultivation of normal rat kidney cells (NRK-49F) on a soft-agar medium followed the method of Roberts *et al.* (1980). To the 35 mm well of a six-well culture plate (Falcon no. 3046), a basal layer of 1.0 ml DMEM in which 10% FCS had been mixed with 0.5% agar was added and hardened. To the layer above this basal layer, $9 \times 10^3$ NKR-49F cells in 1.0 ml of DMEM containing 0.3% agar, 2.5 ng $ml^{-1}$ epidermal growth factor (Nakarai, Tokyo, Japan) and 1 ng $ml^{-1}$ TGF-β1 or 0.1 ml of culture medium or the cell lysate of KATO-III was added and hardened. Culture was continued for 7 days at 37°C under 5% carbon dioxide–air, and the colony number per well was measured.

*Radioreceptor assay*

The radioreceptor assay for TGF-β1 was carried out by the method of Frolik *et al.* (1984). [$^{125}$I]TGF-β1 (specific activity 588 Ci $mmol^{-1}$) was purchased from Amersham Japan (Tokyo, Japan). NRK-49F cells were plated in 24-well culture plates (2.0 $cm^2$ per well, Becton-Dickinson) at a density of $1 \times 10^5$ cells per well. The cells were washed with binding buffer (DMEM pH 7.4 containing 0.1% bovine serum albumin and 25 mM HEPES), and then 1 ml of binding buffer containing 0.25 ng $ml^{-1}$ [$^{125}$I]TGF-β1 and 0.1 ml of samples was added to each well. Non-specific binding of [$^{125}$I]TGF-β1 was determined in the presence of 1 μg of 50% pure unlabelled TGF-β1. The incubation was continued for 2 h at 4°C on a rocker platform. The [$^{125}$I]TGF-β1-bound cells were then washed three times with binding buffer and removed from the plate by treatment with 1 ml of solubilisation buffer (1% Triton

X-100, 10% glycerol, 20 mM HEPES, pH 7.4). Radioactivity of these cells was measured in a gamma-counter. A standard competition curve was constructed with 0.1–100 ng $ml^{-1}$ purified TGF-β1.

*Assay for collagen synthesis by human fibroblasts (HELs)*

We used the method of Postlethwaite *et al.* (1984) to measure the ability of HEL cells to synthesise collagen. HEL cells ($1 \times 10^5$ $ml^{-1}$ per well, 24-well culture plate) were added with 10% KATO-III culture medium containing 250 μg $ml^{-1}$ normal rabbit IgG or 250 μg $ml^{-1}$ anti-TGF-β1 (N92-103) IgG, and 5 μCi $ml^{-1}$ of [$^3$H]proline. After 24 h, protein secreted in the supernatant was precipitated in trichloroacetic acid (TCA), and the amount of [$^3$H]proline incorporated in this protein fraction was determined to give a measure of total protein synthesis. In another experiment the protein in the supernatant was first degraded with bacterial collagenase,

**Figure 1** Immunohistochemical staining of TGF-β1 in gastric carcinomas of various histological types. The cytoplasm of the tumour cells was strongly stained in specimens of scirrhous type (**a**, **b**, **c** and **d**), while in specimens of non-scirrhous type (**e**, **f** and **g**) relatively weak staining was observed. The normal portion of the surgically removed specimen of gastric carcinoma was negative for staining (**h**) (× 170).

and collagenase-insensitive protein was precipitated with TCA, and the amount of [$^3$H]proline incorporated in this precipitate was measured. The amount of [$^3$H]proline incorporated in the collagenase-sensitive fraction was calculated as the amount of [$^3$H]proline incorporated in the total protein fraction minus the amount of [$^3$H]proline incorporated in the collagenase-insensitive fraction. The amount of collagen synthesis relative to total protein synthesis (hereafter designated as the collagen synthesis rate) was calculated by the following equation taken from Peterkofsky and Diegelmann (1971):

$$\text{Collagen synthesis rate (\%)} = \frac{\text{Collagen-sensitive fraction} \times 100}{5.4 \times \text{collagenase-insensitive fraction} + \text{collagenase-sensitive fraction}}$$

### Results

### *Immunohistochemical analysis of TGF-β1 expression in gastric cancer tissues*

Figure 1 shows eight specimens of typical gastric cancer tissues with various histological types stained for TGF-β1. In all four specimens of scirrhous gastric cancer, tumour cells (Figures 1a–d), infiltrating sparsely or forming islets in the interstitial tissue, stained intensely. In the specimens of non-scirrhous cancers (Figure 1e–g), on the other hand, the carcinoma cells stained unevenly and much less intensely than did the scirrhous gastric cancer cells. When staining was as strong as in the scirrhous gastric cancer cells in Figure 1a–d, the grade was defined as (+ +). Similarly, when staining was moderate, as in Figure 1e and f (Borrmann type II gastric cancer), it was graded as (+). Weak staining as in Figure 1g (Borrmann type III gastric cancer) was graded as (±). Failure to stain, as in normal pyloric glands, shown in Figure 1h, was graded as (−). On the basis of such immunohistological observation, the staining intensity of 20 cases of gastric cancer was analysed as shown in Table I. Of the seven scirrhous gastric cancer (Borrmann type IV) tumours (nos. 14–20), one (no. 16) stained moderately (+) and the remaining six stained intensely for TGF-β1 (+ +). In the cases of Borrmann types II and III, non-scirrhous gastric cancers, cancer tissue specimens stained relatively weakly compared with scirrhous cancers. There was a good relationship between the degree of fibrosis and the intensity of staining.

### *TGF-β1 mRNA expression in various gastric cancer cells*

The expression of TGF-β1 mRNA in three different scirrhous gastric cancer cell lines (KATO-III, OCUM-1 and HSC-39) and two different non-scirrhous gastric cancer cell lines (MKN-28 and MKN-45) was investigated by Northern blot analysis. As shown in Figure 2, a band corresponding to TGF-β1 mRNA was observed at 2.5 kb in all the cell lines. The expression of TGF-β1 mRNA signal relative to that of a β-actin signal was 5.8 for KATO-III, 1.6 for OCUM-1, 2.6 for HSC-39, 1.0 for MKN-28 and 0.7 for MKN-45. From these data it is obvious that there was a tendency for the formation of TGF-β1 mRNA to be more marked in scirrhous gastric cancer cells (the first three cases) than in non-scirrhous cancer cells (the last two cases).

### *TGF-β1 activity in the culture media of various gastric cancer cells*

The concentration of TGF-β1 in the culture media of various gastric cancer cell lines was measured by radioreceptor assays, using NRK-49F as a target cell. The results of the measurements are shown in Figure 3a. Without acid treatment, the concentrations of TGF-β1 in the culture media of scirrhous gastric cancer cells were 2.98 ng ml$^{-1}$ for KATO-III, 1.72 ng ml$^{-1}$ for OCUM-1, and 1.63 ng ml$^{-1}$ for HSC-39, while those of non-scirrhous gastric cancer cells (MKN-28 and MKN-45) were below the detection limit. When the measurements were carried out after acid treatment, no change in the concentrations of TGF-β1 was observed for scirrhous cancer cells, but TGF-β1 became detectable in the acid-treated culture media of non-scirrhous gastric cancer cells in concentrations of 1.12 ng ml$^{-1}$ for MKN-28 and 1.54 ng ml$^{-1}$ for MKN-45. In order to determine the intracellular form of TGF-β1, a similar measurement was made on cell lysate from KATO-III. Without acid treatment, the TGF-β1 concentration was below the detection limit, indicating that intracellular TGF-β1 occurs in a latent form while, after acid treatment, it was converted to an active form in a concentration of 2.61 ng ml$^{-1}$.

**Table I** Comparison of the intensity of immunohistochemical staining for TGF-β1 in 20 gastric carcinomas of various histological types

| *Case no.* | *Borrmann classification* | *Histological diagnosis*[a] | *Grade of immunoreactivity*[b] | *Relative amount of fibrous septa* |
|---|---|---|---|---|
| 1 | II | Tub 2 | + | Intermediate |
| 2 | II | Tub 2 | ± | Intermediate |
| 3 | II | Por | + | Intermediate |
| 4 | II | Tub 1 | ± | Intermediate |
| 5 | II | Tub 1 | + | Intermediate |
| 6 | III | Tub 2 | + | Intermediate |
| 7 | III | Tub 2 | + + | Intermediate |
| 8 | III | Tub 1 | + | Intermediate |
| 9 | III | Pap | ± | Medullary |
| 10 | III | Por | + | Intermediate |
| 11 | III | Pap | ± | Medullary |
| 12 | III | Por | + | Intermediate |
| 13 | III | Tub 2 | + | Intermediate |
| 14 | IV | Por | + + | Scirrhous |
| 15 | IV | Sig | + + | Scirrhous |
| 16 | IV | Por | + | Scirrhous |
| 17 | IV | Por | + + | Scirrhous |
| 18 | IV | Por | + + | Scirrhous |
| 19 | IV | Sig | + + | Scirrhous |
| 20 | IV | Por | + + | Scirrhous |

[a]Tub 1, well-differentiated tubular adenocarcinoma; tub 2, moderately differentiated tubular adenocarcinoma; por, poorly differentiated tubular adenocarcinoma; pap, papillary adenocarcinoma; sig, signet-ring cell carcinoma. [b]Grade of immunoreactivity was defined from intensity of staining in Figure 1 as described in the text.

Colony assays for TGF-β1 activity are also carried out to confirm the above observation (Figure 3b). The culture media from scirrhous gastric cancer cells showed apparent colony formation regardless of acid treatment, while colony formation was observed in the culture media of non-scirrhous gastric cancer cells only after acid treatment. The cell lysate of KATO-III did not stimulate colony formation unless it was treated with acid.

**Figure 2** Northern blot analysis of TGF-β1 mRNA from five established gastric carcinoma cell lines. Eight micrograms of total RNA was separated on 1.0% agarose gel, transferred onto nylon filter and hybridised with $^{32}$P-labeled TGF-β1 cDNA probe. A β-actin probe was employed as internal standard. Lane 1, KATO-III; lane 2, OCUM-1; lane 3, HSC-39; lane 4, MKN-28; lane 5, MKN-45.

**Figure 3** TGF-β1 activity in the culture media from five established gastric carcinoma cell lines. The activities were estimated by radioreceptor assay (**a**) and colony assay (**b**) as described in Materials and methods. In both figures, the open and hatched histograms represent the latent and active forms of TGF-β1 respectively.

*Effects of KATO-III culture medium on collagen synthesis by fibroblasts (HELs)*

Collagen synthesis by HEL cells showed an about 6.3-fold increase with the addition of KATO-III cell culture medium (10%), compared with the level of collagen synthesis without the addition of the same culture medium, and the rate of synthesis of collagen was also increased from 6.3% to 34.2%. Furthermore, when the anti-N92-103 antibody with neutralising activity for TGF-β1 was added to the culture medium at a concentration of 250 μg ml$^{-1}$, the increase in collagen synthesis was inhibited by approximately 42% (Figure 4).

### Discussion

In an attempt to clarify the mechanism of the increase in collagen deposition in the stroma of scirrhous gastric carcinoma, the present investigation examined the expression of TGF-β1 in scirrhous gastric carcinoma tissue and the activity of TGF-β1 in cell lines obtained from tissue.

On immunohistochemical analysis, scirrhous gastric cancer cells stained for TGF-β1 more intensely than did non-scirrhous gastric cancer cells, and there was a linear relation-

**Figure 4** Effect of culture medium from KATO-III cells on the collagen synthesis of HEL cells. Collagen synthesis of HEL cells treated with 10% culture medium of KATO-III was measured as described in Materials and methods then 250 μg ml$^{-1}$ anti-N92-103 antibody, which has neutralising activity for TGF-β1, was added to this culture medium to verify the effect of TGF-β1 on collagen synthesis. As a control, normal rabbit IgG was used. Bars indicate mean ± s.d. *$P<0.01$.

ship between staining of TGF-$\beta$1 and the degree of fibrosis in stroma. Furthermore, scirrhous gastric cancer cell lines (KATO-III, OCUM-1 and HSC-39) had a more intense signal of TGF-$\beta$1 mRNA than non-scirrhous gastric cancer cell lines (MKN-28 and MKN-45). Those findings are compatible with the report by Yoshida *et al.* (1989), who found increased expression of TGF-$\beta$1 mRNA in scirrhous cancer tissues, and that by Hirayama *et al.* (1992), who found intense staining of TGF-$\beta$1 in penetrating cells of the linitis plastica type of gastric cancer: they are also consistent with the notion that cancer cells produce TGF-$\beta$1 to promote interstitial proliferation around them. On the other hand, Mizoi *et al.* (1993) recently claimed that despite the fact that both tumour cells and stromal cells were stained positively, fibrosis in gastrointestinal cancer is promoted by TGF-$\beta$1, mainly secreted from stromal cells, which was thought to be the case because latent TGF-$\beta$1-binding protein (LTBP), which is supposed to facilitate the secretion of TGF-$\beta$1, was identified only in stromal cells.

However, lack of LTBP expression in tumour cell does not necessarily mean that tumour cells are unable to secrete TGF-$\beta$1, since cells with no LTBP are also known to secrete TGF-$\beta$1 albeit slowly (Miyazono *et al.*, 1991). An even more important observation was that scirrhous cell lines secreted mainly active TGF-$\beta$1, while non-scirrhous cells secreted latent TGF-$\beta$1. In fact the present studies on established cell lines clearly demonstrated that TGF-$\beta$1 was indeed secreted in the culture medium in an appreciable amount. This finding led us to the postulate that the difference in the amount of interstitial connective tissue in scirrhous and non-scirrhous gastric cancers depends not only on the difference in the amount of TGF-$\beta$1 produced and released but also on whether or not TGF-$\beta$1 is activated.

The next subject of interest was the mechanism by which active TGF-$\beta$1 is released. This was investigated in scirrhous gastric cancer KATO-III cells, which showed the highest TGF-$\beta$1 activity of all tested cancer cell lines. Because TGF-$\beta$1 in the cell lysate was inactive, it is presumed that TGF-$\beta$1 is activated after release from the cancer cell. Subsequent unpublished studies by us showed that scirrhous gastric cancer KATO-III cells secrete a proteinase that converts TGF-$\beta$1 from an inactive to an active form.

A confirmative investigation was then carried out to determine whether or not the culture medium of KATO-III cells promotes collagen synthesis by fibroblasts. The results obtained using the neutralising antibody clearly indicated that TGF-$\beta$1 is one of the factors stimulating collagen synthesis in the culture medium.

Lastly, one may consider the possibility that active TGF-$\beta$1 inhibits the proliferation of tumour cells by an autocrine action. Yanagihara and Tsumuraya (1992), in fact, reported that HSC39 cells underwent apoptosis in the presence of active TGF-$\beta$1. However, their experiment was performed under exceptional conditions; they selected particular clones grown in serum-starved medium. The tumour cells, including HSC39, examined in the present investigations are all established cell lines and therefore apparently are able to proliferate regardless of the fact that some of them produce active TGF-$\beta$1. In addition, it is well known that tumour cells quite often escape from growth suppression by TGF-$\beta$1. The mechanisms of that escape are presently under investigation.

In conclusion, the interstitium in scirrhous gastric cancer seems to proliferate by a mechanism whereby TGF-$\beta$1 secreted from the gastric cancer cells stimulates collagen synthesis by fibroblasts around them.

We previously reported that myelofibrosis in megakaryoblastic leukaemia is attributable to the action of active TGF-$\beta$1 (Terui *et al.*, 1990). Further studies need to be carried out to conclude that active TGF-$\beta$1 is required to produce the vigorous fibrosis common to many malignant tumours.

We wish to thank Dr Irving Listowsky, Albert Einstein College of Medicine, Yeshiva University, for his help in the preparation of this manuscript. This work was supported by a Grant-in-Aid for Cancer Research from the Ministry of Education, Science and Culture of Japan and by grants from the Ministry of Health and Welfare of Japan.

### References

ASSOIAN, R.K., KOMORIYA, A., MEYERS, C.A., MILLER, D.M. & SPORN, M.B. (1983). Transforming growth factor-$\beta$ in human platelets. *J. Biol. Chem.*, **258**, 7155–7160.

BORRMANN, R. (1926). Geschwülste des Magens und Duodenums. In *Handbuch der speziellen pathologischen Anatomie und Histologie*, Vol. IV/1, Hanke, F. & Lubarsch, O. (eds) p. 865. Springer: Berlin.

CHOMCZYNSKI, P. & SACCHI, N. (1987). Single-step method of RNA isolation by acid guanidinium thiocyanate-phenol-chloroform extraction. *Anal. Biochem.*, **162**, 156–159.

FEINBERG, A.P. & VOGELSTEIN, B. (1984). A technique for radiolabelling DNA restriction endonuclease fragments to high specific activity. *Anal. Biochem.*, **137**, 266–267.

FROLIK, C.A., WAKEFIELD, L.M., SMITH, D.M. & SPORN, M.B. (1984). Characterization of a membrane receptor for transforming growth factor-$\beta$ in normal rat kidney fibroblasts. *J. Biol. Chem.*, **259**, 10995–11000.

GOLDBERG, D.A. (1980). Isolation and partial characterization of Drosophila alcohol dehydrogenase gene. *Proc. Natl Acad. Sci. USA*, **77**, 5794–5798.

HEINE, U.I., MUNOZ, E.F., FLANDERS, K.C., ELLINGSWORTH, L.R., LAM, H.Y.P., THOMPSON, N.L., ROBERTS, A.B. & SPRON, M.B. (1987). Role of transforming growth factor-$\beta$ in the development of the mouse embryo. *J. Cell Biol.*, **105**, 2861–2876.

HIRAYAMA, D., FUJIMORI, T., SATONAKA, K., NAKAMURA, T., KITAZAWA, S. & HORIO, M. (1992). Immunohistochemical study of epidermal growth factor and transforming growth factor-$\beta$ in the penetrating type of early gastric cancer. *Hum. Pathol.*, **23**, 681–685.

IGNOTZ, R.A. & MASSAGUE, J. (1986). Transforming growth factor-$\beta$ stimulates the expression of fibronectin and collagen and their incorporation into the extracellular matrix. *J. Biol. Chem.*, **261**, 4337–4356.

KUBO, T. (1991). Establishment and characterization of a new gastric cancer cell line (OCUM-1), derived from Borrmann type 4 tumor. *Jpn. J. Surg.*, **92**, 1451–1460.

MIYAZONO, K., OLOFSSON, A., COLOSETTI, P. & HELDIN, C.H. (1991). A role of the latent TGF-$\beta$1-binding protein in the assembly and secretion of TGF-$\beta$1. *EMBO J.*, **10**, 1091–1101.

MIZOI, T., OHTANI, H., MIYAZAWA, M., MATSUNO, S. & NAGURA, H. (1993) Immunoelectron microscopic localization of transforming growth factor $\beta$1 and latent transforming growth factor $\beta$1 and latent transforming growth factor $\beta$1 binding protein in human gastrointestinal carcinomas: qualitative difference between cancer cells and stromal cells. *Cancer Res.*, **53**, 183–190.

NAGAYO, T., OHMORI, Y., OKAJIMA, K., SANO, R., SUGANO, H., SUZUKI, H., SENOO, T., FUKUSHIMA, K., TANAKA, N., NAGATOMO, T., NISHI, M., MIWA, K. & MOCHIZUKI, T. (1985). Histological classification of gastric cancer. In *The General Rules for the Gastric Cancer Study*, 11th edn., Japanese Research Society for Gastric Cancer (ed.) pp. 41–75. Kanehara Press: Tokyo.

NIITSU, Y., ITO, N., KOHDA, K., OWADA, M., MORITA, K., SATO, S., WATANABE, N., KOHGO, Y. & URUSHIZAKI, I. (1988*a*). Immunohistochemical identification of type I procollagen in tumour cells of scirrhous adenocarinoma of the stomach. *Br. J. Cancer*, **57**, 79–82.

NIITSU, Y., URUSHIZAKI, Y., KOSHIDA, Y., TERUI, K., MAHARA, K., KOHGO, Y. & URUSHIZAKI, I. (1988*b*). Expression of TGF-beta gene in adult T cell leukemia. *Blood*, **71**, 263–266.

O'CONNOR-MCCOURT, M.D. & WAKEFIELD, L.M. (1987). Latent transforming growth factor-$\beta$ in serum. *J. Biol. Chem.*, **262**, 14090–14099.

PETERKOFSKY, B. & DIEGELMANN, R. (1971). Use of a mixture of proteinase-free collagenases for the specific assay of radioactive collagen in the presence of other proteins. *Biochemistry*, **10**, 988–994.

POSTLETHWAITE, A.E., SMITH, G.N., MAINARDI, C.L., SEYER, J.M. & KANG, A.H. (1984). Lymphocyte modulation of fibroblast function in vitro: stimulation and inhibition of collagen production by different effector molecules. *J. Immunol.*, **132**, 2470–2477.

ROBERTS, A.B., LAMB, L.C., NEWTON, D.L., SPORN, M.B., DELARCO, J.E. & TODARO, G.J. (1980). Transforming growth factors: Isolation of polypeptides from virally and chemically transformed cells by acid/ethanol extraction. *Proc. Natl Acad. Sci. USA*, **77**, 3494–3498.

SEKIGUCHI, M., SASAKIBARA, K. & FUJI, G. (1978). Establishment of cultured cell lines derived from a human gastric carcinoma. *Jpn. J. Exp. Med.*, **48**, 61–68.

TERUI, T., NIITSU, Y., MAHARA, K., FUJISAKI, Y., URUSHIZAKI, Y., MOGI, Y., KOHGO, Y., WATANABE, N., OGURA, M. & SAITO, H. (1990). The production of transforming growth factor-$\beta$ in acute megakaryoblastic leukemia and its possible implications in myelofibrosis. *Blood*, **75**, 1540–1548.

URUSHIZAKI, Y., NIITSU, Y., TERUI, T., KOSHIDA, Y., MAHARA, K., KOHGO, Y., URUSHIZAKI, I., TAKAHASHI, Y. & ITO, H. (1987). Cloning and expression of the gene for human transforming growth factor-$\beta$ in Escherichia coli. *Tumor Res.*, **22**, 41–55.

YANAGIHARA, K. & TSUMURAYA, M. (1992). Transforming growth factor $\beta$1 induces apoptotic cell death in cultured human gastric carcinoma cells. *Cancer Res.*, **52**, 4042–4045.

YANAGIHARA, K., SEYAMA, T., TSUMURAYA, M., KAMADA, N. & YOKORO, K. (1991). Establishment and characterization of human signet ring cell gastric carcinoma cell lines with amplification of the c-myc oncogene. *Cancer Res.*, **51**, 381–386.

YOSHIDA, K., YOKOZAKI, H., NIMOTO, M., ITO, H,. ITO, M. & TAHARA, E. (1989). Expression of TGF-$\beta$ and procollagen type I and type III in human gastric carcinomas. *Int. J. Cancer*, **44**, 394–398.